# Pair formation, home range, and spatial variation in density, size and social status in blotched foxface *Siganus unimaculatus* on an Okinawan coral reef

**DOI:** 10.7717/peerj.1280

**Published:** 2015-09-24

**Authors:** Atsushi Nanami

**Affiliations:** Research Center of Sub-tropical Fisheries, Seikai National Fisheries Research Institute, Fisheries Research Agency, Ishigaki, Okinawa, Japan

**Keywords:** *Siganus unimaculatus*, Rabbitfish, Size-assortative pairing, Overlapping territory, Ontogenetic habitat shift, Pair formation

## Abstract

The present study examined pair formation, spatial pattern of home range and spatial variation in density, size and social status of blotched foxface *Siganus unimaculatus* (family Siganidae) on an Okinawan coral reef. Of 32 pairs sampled for sexing, 31 (96.9%) were heterosexual and showed size-assortative pairing. Developed ovaries were found in April and July, whereas oocytes were immature in August, September and February. Heterosexual pairing was found in both reproductive and non-reproductive periods. Home range size tended to be positively related to fork length (FL). The degree of home range overlap for same size class pairs was smaller than that for different size class pairs. The intraspecific behavior when two pairs approached each other was categorized as ‘attack,’ ‘agonistic display’ and ‘no interactions,’ and the frequency of agonistic behaviors (“attack” or “agonistic display”) was significantly greater than “no interactions.” Underwater observations at a seagrass bed, a rocky reef flat and a sheltered reef slope revealed that the mean FL was significantly smaller at the sheltered reef slope (4–13 cm) than at the rocky reef flat (>13 cm). No individuals were found in the seagrass bed. Most individuals less than 6 cm FL were solitary, whereas most individuals over 7 cm FL were paired. Density was significantly greater on the sheltered reef slope than on the rocky reef flat.

## Introduction

Coral reefs support a very high diversity of fish species and clarifying the ecological and behavioral aspects leading to the high species richness on coral reefs is central to understanding coral reef ecosystems. Among diverse fish species, herbivores (e.g., parrotfishes and surgeonfishes) are considered to be important for the maintenance of a healthy ecosystem balance in coral reefs (Hughes, 1994; Bellwood, Hughes & Hoey, 2006; Hughes et al., 2007). Recently, rabbitfish (family Siganidae) have also been suggested as important ecological members to maintain the ecosystem balance through their herbivorous feeding behavior (Fox & Bellwood, 2007; Fox & Bellwood, 2013; Hoey, Brandl & Bellwood, 2013). There is relatively little information available on basic aspects of siganid behavior such as social systems, home ranges, reproduction and ontogenetic shifts in habitat use. However, several previous studies have shown that some siganid species inhabiting coral reefs form pairs (Woodland, 1990; Brandl & Bellwood, 2013; Brandl & Bellwood, 2014; Fox & Donelson, 2014). Brandl & Bellwood (2013) have shown for *Siganus doliatus* that 75% of pairs are heterosexual pairs, whereas 25% of pairs are same sex pairs. Fox & Donelson (2014) have suggested that pairs of individuals of *S. vulpinus*, *S. corallinus* and *S. puellus* show coordinated vigilance against predators. These results have highlighted the potential for pair formation in rabbitfish to be based on factors such as feeding behavior and/or predator avoidance, rather than just reproduction (Brandl & Bellwood, 2014; Fox & Donelson, 2014). However, details of the behavioral aspects of pair formation for siganid species have not been sufficiently studied yet.

Home range size and its spatial arrangement are important aspects for behavioral ecology of coral reef fish for determining the appropriate size of marine protected areas, and many studies have examined the home range size of various coral reef fishes (e.g., Kramer & Chapman, 1999; Zeller, 1997; Meyer & Holland, 2005; Taylor & Mills, 2013). In contrast, the home range sizes of siganid species are known only *S. lineatus* (Fox & Bellwood, 2011) and *S. doliatus* (Brandl & Bellwood, 2013). The size and spatial arrangement of home ranges of marine fishes are often related to territoriality (Yabuta, 1997; Kokita & Nakazono, 1999; Matsumoto, 2001; Nanami & Yamada, 2008). These studies have shown that the each individual or pair for the species has clear home range boundaries that are defended by attacking and agonistic display. However, few studies have examined the home range defending behavior of siganid species in relation to the spatial pattern of home ranges.

Clarifying the characteristics of habitat use of marine organisms is also essential to provide effective management. Numerous previous studies have shown the size-specific spatial distribution of coral reef fishes (Brokovich et al., 2007; Claisse et al., 2009; Nagelkerken, 2009). For example, juveniles and adults show different habitat distribution for some coral reef fishes (Nagelkerken, 2009). Although some studies have shown the spatial distribution of siganid species among various habitat zones (Hoey, Brandl & Bellwood, 2013; Brandl & Bellwood, 2013), there have been no studies that show spatial variation in density, size and pairing pattern for siganids.

Blotched foxface *Siganus unimaculatus* is commonly found on coral reefs of the western Pacific including Okinawan coral reef (Allen et al., 2003) and usually found in pairs (Masuda & Kobayashi, 1994; Carpenter & Niem, 2001). Although Kuriiwa et al. (2007) demonstrated by phylogenetic analysis that the genetic distance between *S. unimaculatus* and *S. vulpinus* is very small, *S. unimaculatus* is treated as a different species from *S. vulpinus* in the present study.

The aim of this study was to present a detailed description of the social demography of *Siganus unimaculatus* from Okinawa, in particular, aspects pertaining to pair formation, home range size and overlap, ontogentic habitat usage and agonistic behavior. Specifically, based on visual observations on an Okinawan coral reef, the study aimed at answering the following questions: (1) Are pairs of *S. unimaculatus* heterosexual? (2) Does the pair formation have any specific ecological traits such as size-assortative pairing and sexual difference in size? (3) Is there any particular spatial pattern of the home ranges? (4) Are intra-specific interactions such as agonistic behavior found in relation to territoriality? (5) Are there any spatial variations in density and size? (6) Is there any size-related difference in social status?

## Materials and Methods

The study was conducted mainly using field observations of free-living fishes in their natural habitat. Individuals caught for sampling were immediately killed by placing them on ice in order to minimize pain. The sampling procedure was approved by Okinawa prefectural government fisheries coordination regulation No. 41, which permits capture of marine fishes on Okinawan coral reefs for scientific purposes.

### Sex of paired individuals

**Figure 1:**
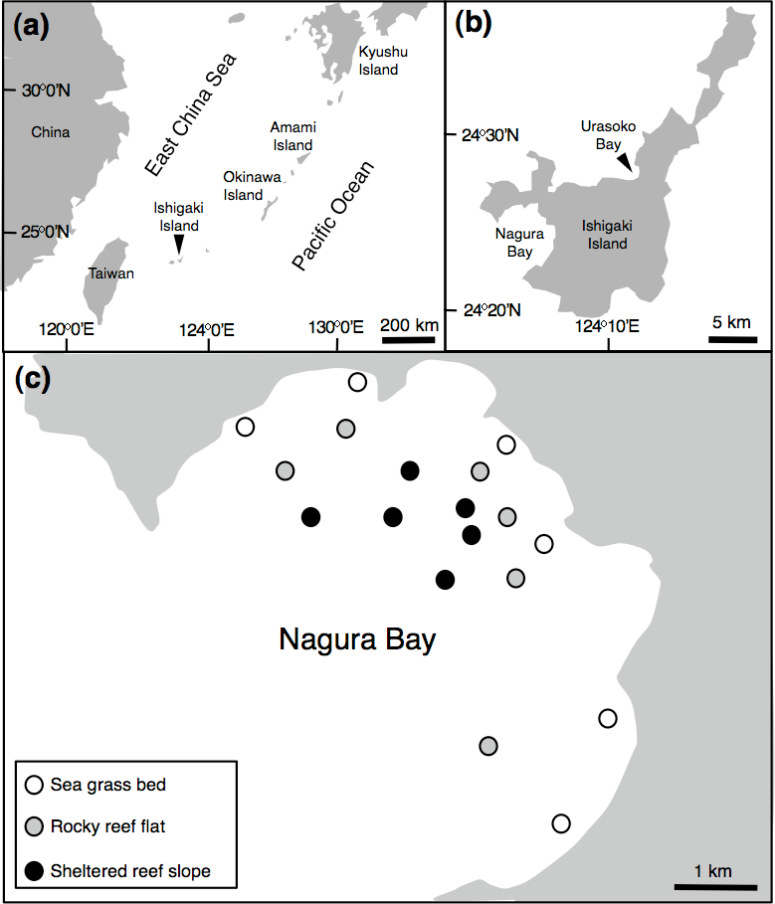
Map of the study site. Showing the position of Ishigaki Island (A), Urasoko Bay and Nagura Bay (B) and study sites for estimating *Siganus unimaculatus* density in Nagura Bay (C).

In order to identify the sex of paired individuals, 32 pairs were collected around Ishigaki Island (Fig. 1) using a spear gun between April 2013 and February 2014 (April (6 pairs), July (12 pairs), August (2 pairs), September (2 pairs), February (10 pairs)). Fork length (FL) ranged from 123.0 mm to 207.5 mm. All specimens were measured for FL (nearest 0.5 mm), whole body mass (g) and gonad mass (nearest 0.01 g). In order to determine sex, gonads were placed in 20% buffered formalin for 48 h, then transferred to 70% ethanol. After dehydration using an ethanol series, gonads were embedded in paraffin, serially sectioned at 6 µm, stained with Mayer’s hematoxylin-eosin and examined using a microscope. For females, oocyte development was classified into six stages, peri-nucleolus, oil-droplet, primary yolk, secondary yolk, tertiary yolk, and mature oocytes. The relationship between FL and whole body mass is shown in Fig. S1.

Potential size differences between sexes within individual pairs were examined using Wilcoxon signed-ranks tests. The relationship between male and female FL within pairs (degree of size-assortative pairing) was examined using a Pearson correlation.

### Spatial pattern of home ranges

**Figure 2:**
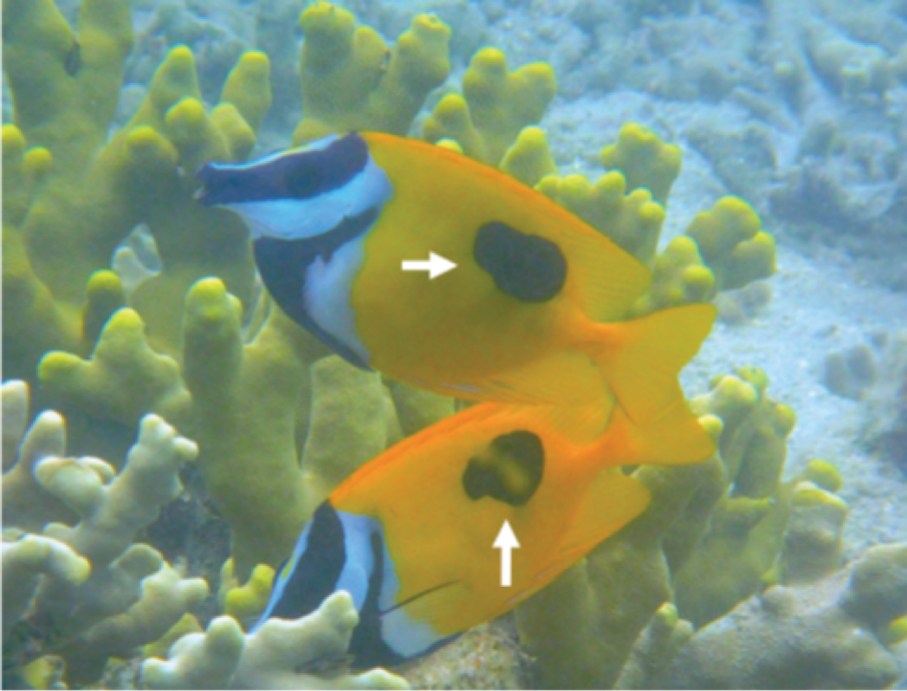
Lateral view of Siganus unimaculatus pair. Showing the black spots that were used for individual identifications (white arrows).

In order to determine the spatial pattern of home ranges, visual observations were conducted in a 40 m × 40 m quadrat at a depth of about 3 m on the fringing reef of Urasoko Bay on Ishigaki Island, Okinawa, Japan (Fig. 1B). The study site was covered by branching *Porites cylindrica*, coral rubble and sand. The procedure of the observations was in accordance with Nanami & Yamada (2008). During June to August 2014, all *Siganus unimaculatus* in the quadrat were identified individually according to the shape of the black spots on each side of the body (Fig. 2). The shapes of the two spots were sketched on waterproof paper while snorkeling. The spot shapes were checked for each individual at each observation. Individuals were readily differentiated based on this pigment pattern, which showed minimal change over the study period. Twelve pairs were observed in the quadrat. Their FL was estimated underwater to the nearest 0.5 cm (ranged: 10.5 cm to 17.5 cm) by reference to a small ruler.

Since Fox & Bellwood (2011) have shown that the active period (diurnal or nocturnal) of *Siganus lineatus* may differ between populations, the active period of *S. unimaculatus* was checked at Urasoko Bay. Preliminary observations revealed that this species was active only during the day and became inactive and changed color during the night (Fig. S2). Therefore, visual observations for home range estimations were made during the day (between 09:00 and 17:30 h). During the visual observations, a GPS receiver sealed in a waterproof case was kept in a box made of styrene foam and towed on the surface. The researcher (A Nanami), using snorkeling gear, slowly approached a pair and followed the fish, taking care to minimize any disturbance. Each pair was followed for 20 min and their position was recorded at 1-s intervals. This procedure was repeated 6 times for each pair.

To estimate the shape and boundaries of each home range, the data logged by the GPS receiver were plotted for each pair using MapSource software (GARMIN). The data from the six swimming track surveys that were obtained for each pair were pooled. Then, the edge of the pooled-tracking data was enclosed and regarded as the ‘home range.’ The boundaries of the home range could be reliably estimated from the 6 sets of observation data (Figs. S3 and S4). The projected areas of all home ranges were measured on a Macintosh computer using an NIH image program (developed at the US National Institutes of Health and available on the Internet at http://rsb.info.nih.gov/nih-image/).

The relationship between home range size and FL of home range owners was obtained as follows: log10 (home range size: m^2^) = a log10 (FL: cm) + *b*; where *a* and *b* are coefficients. The degree of home range overlap was calculated for each home range owner as follows: ((total area overlapping with the home ranges of other pairs) / (home range area of the owner)). The size of the pair was averaged and the average FL was used for the analysis. The average FL values were divided into four arbitrary size classes (class 1: 10 cm–11.5 cm; class 2: 12 cm–13.5 cm; class 3: 14 cm–15.5 cm; class 4: 16 cm–17.5 cm) for subsequent data analyses. One-way analysis of variance (ANOVA) was carried out to assess whether the degree of home range overlap of the same size class pairs was significantly different to that of different size class pairs. For this analysis, the values of home range overlap were arc-sine transformed to ensure the assumption of normality.

### Intraspecific interactions

Intraspecific interactions were recorded in Urasoko Bay while following each pair by snorkeling with the GPS receiver. The observer recorded each occurrence of: (1) attack (rushing at other conspecific pairs and driving the other pair outside of the home range), (2) agonistic display (approaching other conspecific pairs and showing fin displays such as raising the dorsal, pelvic and anal fins) and (3) no interaction (no noticeable change in behavior when pairs were within 50 cm of each other). Each behavior was designated as: (a) directed towards the other pair by the focal pair or (b) directed towards the focal pair by another pair.

The number of three types of the behaviors (during 20-min observation period) was calculated for each pair. Since six sets of 20-min observations were conducted as mentioned above, average number of behaviors was used for each pair. Both ‘attack’ and ‘agonistic display’ were regarded as ‘agonistic behavior.’

### Spatial variation in density, size and social status

In order to examine the spatial variation in density, size and social status of the species, underwater observations were conducted during August and September 2013 at Nagura Bay (Fig. 1C). Six sites were established in each of three habitat types: seagrass beds (water depth = 1–2 m), rocky reef flats (water depth = 2–4 m) and sheltered reef slope (water depth = 10–12 m) (Fig. 1C). The substrate characteristics for the three habitat types were as follows: seagrass bed sites were mostly covered by *Cymodocea serrulata* (Nanami, Asami & Chimura, 2009); rocky reef flat sites were covered by branching *Acropora*, tabular *Acropora*, massive *Porites* and coral rubble (A Nanami, 2015, unpublished data); the sheltered reef slope site were covered by staghorn *Acropora*, bottlebrush *Acropora* and coral rubble (Nanami et al., 2013). At each site, four 50 m × 4 m line transects, separated by more than 20 m, were set. Underwater visual censuses were conducted either by snorkeling or SCUBA. FL and social status of censused individuals were simultaneously recorded. FL was estimated visually by reference to a small ruler carried during the underwater observations. The social status was simultaneously categorized as ‘solitary,’ ‘pair’ or ‘conspecific aggregation.’

Differences in density between habitat types were examined using a Mann–Whitney U-test. Differences in body size distribution were examined via a Kolmogorov–Smirnov test, after pooling the six sites in each habitat. For individuals found as pairs, size similarity between partners was examined using Pearson correlation.

## Results

### Sex identification and sexual difference in size

**Figure 3:**
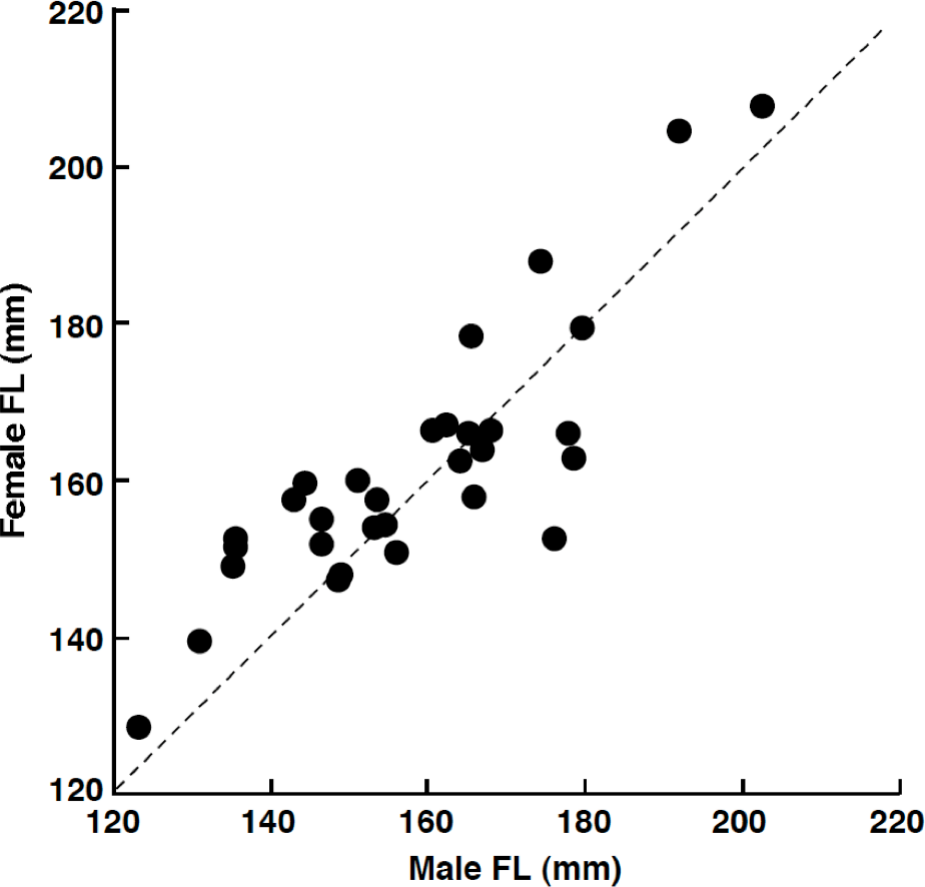
The relationship between male fork length (FL) and female FL for heterosexual pair individuals of *Siganus unimaculatus*. The dotted line represents the case for male FL is equal to female FL.

Among the 32 pairs, 31 pairs were heterosexual and 1 pair was male–male (Table 1). Developed ovaries (tertiary yolk stage and mature oocytes) were found in April (3 of 5 females) and July (4 of 12 females) whereas oocytes were immature (peri-nucleolus stage or oil-droplet stage) for all individuals in August (2 females), September (2 females) and February (10 females). Heterosexual pairs were found in both reproductive (April and July) and non-reproductive (August, September and February) periods (Table 1). Of the heterosexual pairs, the FL of females was larger than that of males in 19 pairs (Table 1). Overall, FL of females was significantly greater than that of males (Wilcoxon signed-ranks test, *p* < 0.05; *n* = 31). The average size difference between males and females of the same pair was 3.3 mm FL (±9.6 standard deviation: SD; *n* = 31). There was a significant positive relationship between male FL and female FL for heterosexual pairs (Pearson correlation, *r* = 0.850, *p* < 0.01) (Fig. 3).

**Table 1:** Size, gonadosomatic index (GSI) and ovarian development stage in relation to sampling date for 32 pairs of *Siganus unimaculatus*. Ovarian developmental stages are abbreviated. Stages marked with an asterisk (∗) are regarded as “actively reproducing.”

		FL (mm)				
Sampling date	Pair no.	Male	Female	Female GSI	Ovary developmental stage	FL difference (mm) (male–female)
April 2013	1	165.5	178.5	2.86	TYS*	−13.0
	2	174.5	188.0	3.59	TYS*	−13.5
	3	179.5	179.5	3.03	TYS*	0.0
	4	176.0	152.5	0.74	ODS	23.5
	5	148.5	147.5	0.91	PNS	1.0
July 2013	6	165.0	166.0	29.58	MA*	−1.0
	7	153.5	157.5	0.56	PNS	−4.0
	8	178.0	166.0	15.96	MA*	12.0
	9	154.5	154.5	0.54	PNS	0.0
	10	143.0	157.5	0.49	PNS	−14.5
	11	135.5	152.5	0.97	PYS*	−17.0
	12	146.5	155.0	1.06	PNS	−8.5
	13	144.5	159.5	1.83	TYS*	−15.0
	14	135.0	149.0	0.86	PNS	−14.0
	15	151.0	160.0	1.13	PNS	−9.0
	16	123.0	128.5	0.45	PNS	−5.5
	17	146.5	152.0	0.52	ODS	−5.5
August 2013	18	160.5	166.5	0.78	PNS	−6.0
	19	178.5	163.0	0.55	ODS	15.5
September 2013	20	192.0	204.5	0.69	PNS	−12.5
	21	202.5	207.5	0.69	PNS	−5.0
February 2014	22	156.0	151.0	0.50	PNS	5.0
	23	135.5	151.5	0.43	PNS	−16.0
	24	131.0	139.5	0.38	PNS	−8.5
	25	166.0	158.0	0.64	PNS	8.0
	26	149.0	148.0	0.57	PNS	1.0
	27	153.0	154.0	0.87	PNS	−1.0
	28	168.0	166.5	0.75	PNS	1.5
	29	164.0	162.5	0.56	PNS	1.5
	30	162.5	167.0	0.81	PNS	−4.5
	31	167.0	164.0	0.75	PNS	3.0
		**Male**	**Male**			
April 2013	32	157.0	169.5	–	–	–

**Notes.**

PNS peri-nucleolus stage ODS oil-droplet stage PYS primary yolk stage TYS tertiary yolk stage MA maturation oocytes

### Spatial pattern and size of home ranges

**Figure 4:**
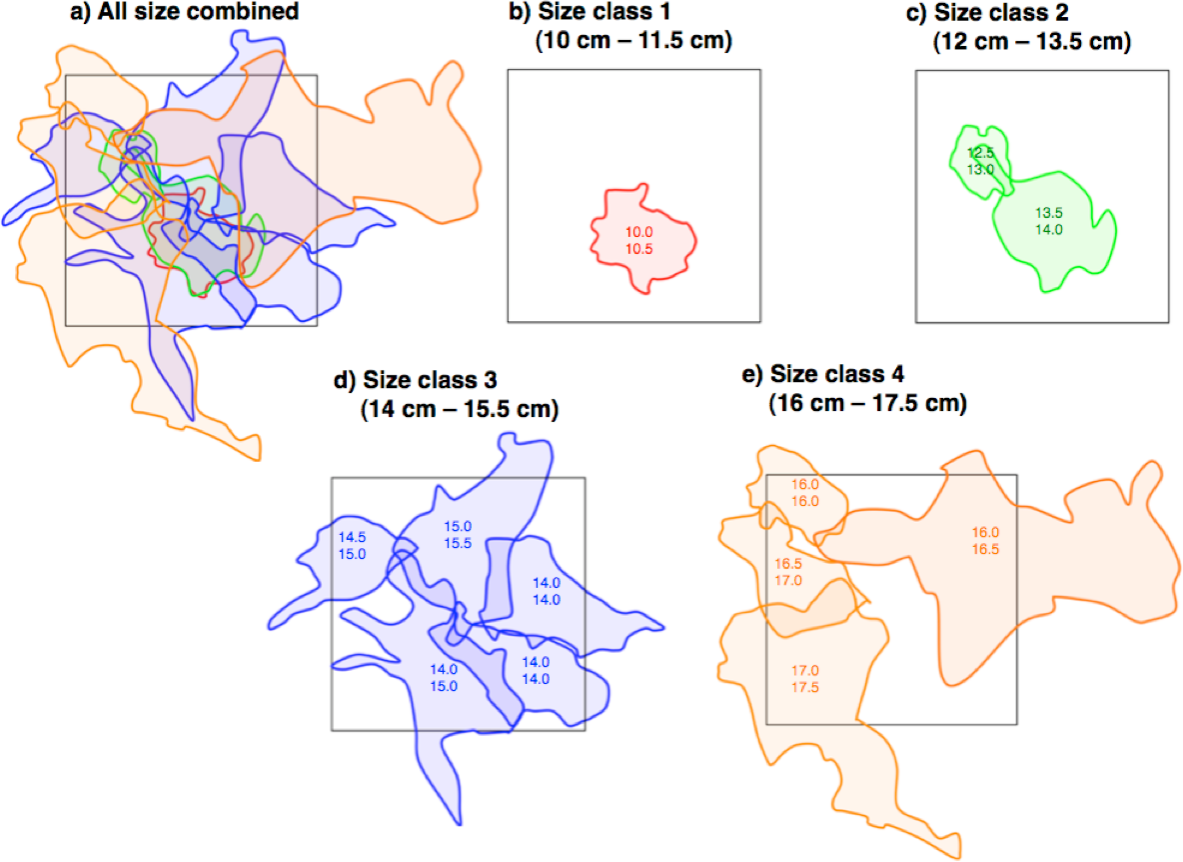
Spatial arrangement of home ranges of *Siganus unimaculatus* in the quadrat (40 m × 40 m: black square). Twelve pairs were found in the quadrat. Areas enclosed by same-colored lines represent the home ranges for same size class pairs. Numbers in areas represent the fork lengths of the pairs (cm).

Twelve pairs were found in the 40 m × 40 m quadrat (Fig. 4A). For size class 1, only one pair was found (Fig. 4B). The home range overlap among same size class pairs was significantly less than that among different size class pairs (15.1% ± 11.4 SD vs. 63.1% ± 26.5 SD) (one-way ANOVA, *F* = 21.8, *df* = 1, *p* < 0.01) (Figs. 4B–4E and Table 2).

**Table 2:** The degree of home range overlap among the four size classes of *Siganus unimaculatus* (% + standard deviation).

Pair size class	No. of pairs	Overlapping with same-sized pairs	Overlapping with different-sized pairs
Size class 1 (10 cm–11.5 cm)	1	0	100
Size class 2 (12 cm–13.5 cm)	2	14.4 ±10.0	93.0 ±9.8
Size class 3 (14 cm–15.5 cm)	5	20.2 ±10.3	66.2 ± 9.2
Size class 4 (16 cm–17.5 cm)	4	12.9 ± 2.5	35.1 ± 17.6
Average		15.1 ± 11.4	63.1 ± 26.4

**Figure 5:**
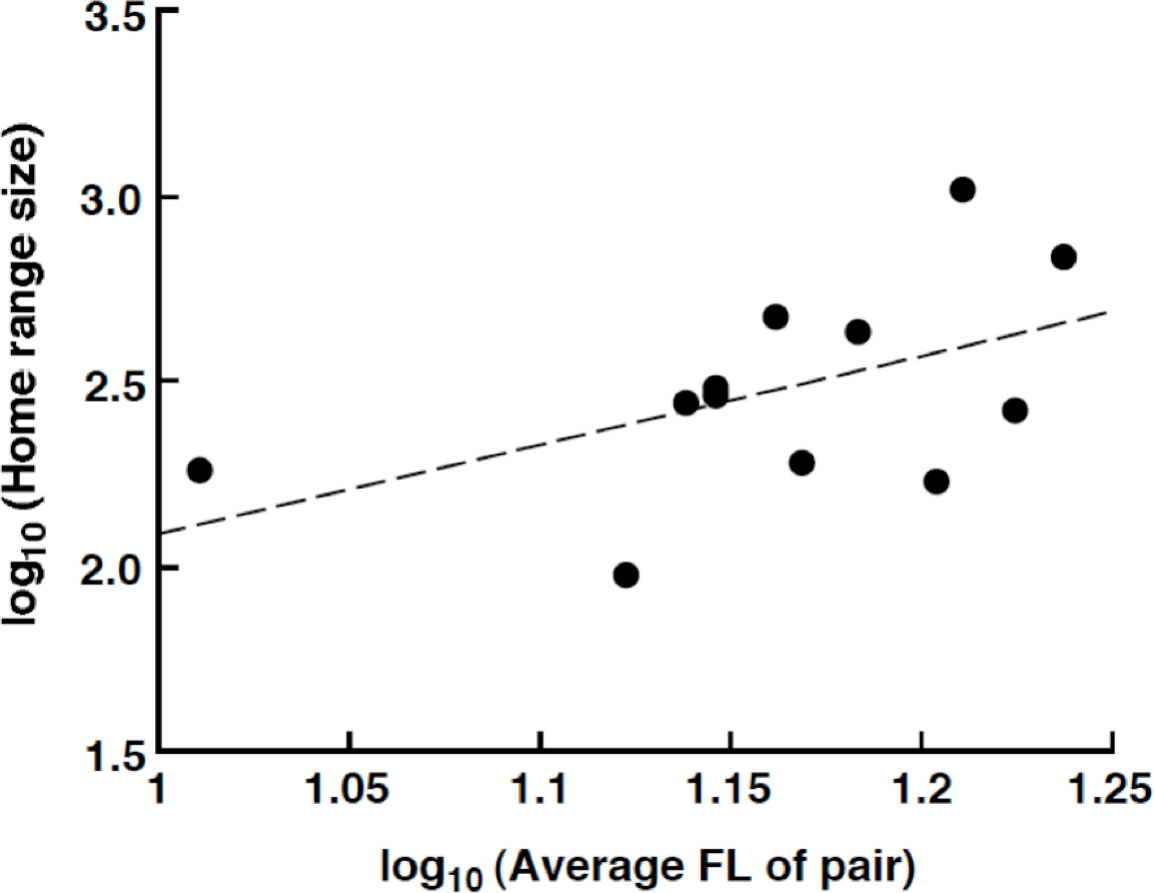
Relationship between the home range size and the average fork length of the *Siganus unimaculatus* pairs.

The home range size varied from 94.5 m^2^ (average FL = 13.25 cm) to 1038.0 m^2^ (average FL = 16.25 cm). There was a nonsignificant trend for a positive relationship between the home range size and average FL of the pair: log_10_ (home range size: m^2^) = 2.40 log_10_ (average FL of pair: cm) – 0.31 (*R*^2^ = 0.257, *p* = 0.093, Fig. 5).

### Intraspecific interactions

The twelve focal pairs performed a mean of 0.42 ± 0.31 SD (range: 0–0.83) attacks, 0.36 ± 0.28 (0–1) agonistic displays and 0.04 ± 0.08 (0–0.17) non-agonistic interactions toward other pairs and received a mean of 0.89 ± 1.06 (0–3.33) attacks, 0.47 ±0.69 (0–2.5) agonistic displays and 0.01 ± 0.05 (0–0.17) non-agonistic interactions from other pairs per 20 min.

### Comparison of density, size and social status among three habitats

No *Siganus unimaculatus* were observed in the seagrass bed. In contrast, a total of 90 individuals per 4,800 m^2^ ((50 m × 4 m) line transect × 4 replicates × 6 sites) were found on the rocky reef flat and 327 individuals per 4,800 m^2^ on the sheltered reef slope. The density of the species (number of individuals per one line transect: 50 m × 4 m) was significantly greater at the sheltered reef slope (13.7 individuals ± 2.9 SD) than at the rocky reef flat (3.8 individuals ± 1.6 SD) (Mann–Whitney U-test, *p* < 0.01).

**Figure 6:**
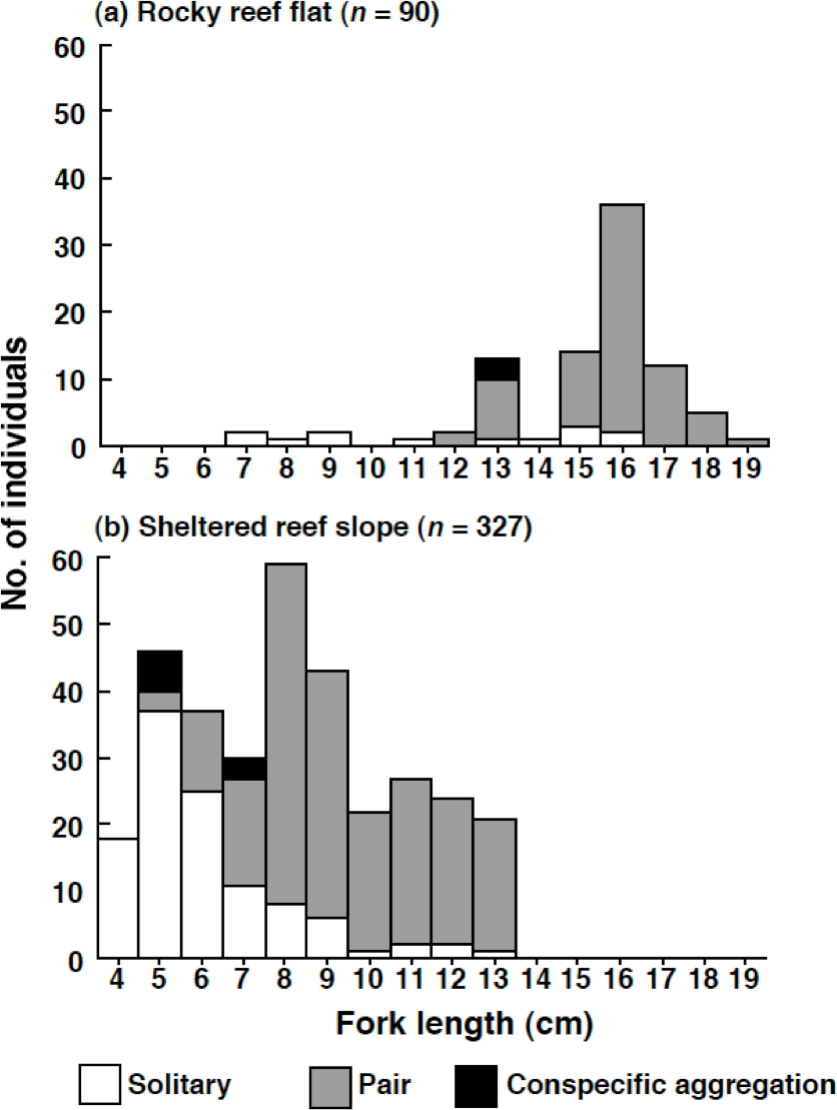
Size distribution of *Siganus unimaculatus* in two habitats. (A) rocky reef flat and (B) sheltered reef slope. The data represent pooled results from six 50 m × 4 m transects in each habitat.

The size of the individuals was significantly larger on the rocky reef flat than on the sheltered reef slope (Kolmogorov–Smirnov test, *χ*^2^ = 209.2, *df* = 2, *p* < 0.05) (Fig. 6). The estimated FL for most individuals on the rocky reef flat was over 13.0 cm. In contrast, the estimated FL for individuals on the sheltered reef slope ranged from 4.0 cm to 13.0 cm, and no individuals over 14.0 cm were found.

On the rocky reef flat, most individuals were found as pairs (Fig. 6). In contrast, on the sheltered reef slope, most individuals less than 5.0 cm (*n* = 64) were found as solitary individuals (*n* = 55, 85.9%); some individuals in between 6.0 and 7.0 cm (*n* = 67) formed pairs on the sheltered reef slope (*n* = 28, 41.8%); most individuals over 8.0 cm (*n* = 196) were found as pairs (*n* = 176, 89.8%).

For all pairs, correlation analysis revealed that the there was a significant positive relationship between FL of smaller-sized individuals and FL of larger-sized individuals (reef flat: *r* = 0.963, *p* < 0.0001; sheltered reef slope: *r* = 0.978, *p* < 0.0001; two habitats combined: *r* = 0.992, *p* < 0.0001) (Fig. S5).

## Discussion

### Pair formation

The present study was the first to examine behavioral aspects of pair formation of *Siganus unimaculatus*. Since developed ovaries were found in April and July, it is suggested that the reproductive season is during April and July which was the first report for the species. Most pairs were heterosexual although there was one same sex pair. Since heterosexual pairs remained in non-reproductive periods, there is a possibility that pair formation is related to home range defense (Whiteman & Côte, 2004). Roberts & Ormond (1992) suggested that heterosexual pairing is found where territory guarding by a pair is more effective than guarding by a solitary individual. Pair formation might increase the success rate of detection and exclusion of intruding pairs. Size-assortative pair formation was also found. Males would prefer larger females due to the larger number of eggs. In addition, if pair formation is effective for home range defense, larger females would provide greater assistance for males. As a result, the larger-sized individuals would require larger-sized partner and size-assortative pair formation will be found. Fox & Donelson (2014) suggested that three species of siganids on coral reefs show coordinated vigilance against predators. However, *S. unimaculatus* did not show coordinated vigilance during feeding, and simultaneous feeding of pairs was observed on an Okinawan coral reef (Video S1). Another possibility for pairing behavior is related to reproduction as most of the pairs were heterosexual. However, pairing behavior was also found for smaller-sized individuals (from 7 cm FL) and these smaller-sized individuals seemed to be still immature, suggesting that reproduction itself may not be the sole factor leading to the initiation of pair formation.

Another reason why size-assortative pair formation is found might be formation stable pair bonds (Pratchett, Pradjakusuma & Jones, 2006). The present study showed that pair formation was found for pairs over 7 cm FL and there were no pairs which had markedly different sizes in FLs with each other. If the pair bond is fairly strong, the size-assortative pairing would be maintained over time.

### Spatial pattern of home ranges

The present study showed that the degree of home range overlap for same-sized pairs is smaller than the overlap for different-sized pairs. Such spatial arrangement has been found for other marine fishes (Matsumoto, 2001; Nanami & Yamada, 2008) and freshwater fishes (Kohda, 2008). These previous studies have shown that the fish species having ‘overlapping territories’ (sensu Matsumoto, 2001; Nanami & Yamada, 2008) are solitary and feed on benthic animals and fishes that allow fishes of different sizes to feed on different prey sizes. In contrast, *S. unimaculatus* forms pairs and is herbivorous (A. Nanami, unpublished data). The mechanisms responsible for overlapping home ranges therefore need to be clarified.

The slight overlap of home ranges for same-sized pairs was consistent with previous studies (Matsumoto, 2001; Nanami & Yamada, 2008). This overlap might represent attempts by some pairs to expand their home range, as indicated by the observed aggregation between pairs. Such intraspecific interaction might be a cause the slight overlap of home ranges at the boundaries.

Most intra-specific interactions with other pairs were aggressive and non-agonistic encounters were rare. This suggests the territoriality of the species, which is probably the first finding for siganids. Since the average frequency of the agonistic behavior (attacks and agonistic display) was not high (less than 1 per a 20-min observation period), each pair might reduce the number of interactions between potential antagonists by subtle avoidance from other pairs. The aggressive behavior might be also reduced toward familiar overlapping pairs.

### Spatial variation in density, size and social status

Some previous studies have found ontogenetic spatial variations in coral reef fishes (e.g., Dorenbosch et al., 2005; Brokovich et al., 2007; Verweij et al., 2008; Claisse et al., 2009; reviewed in Nagelkerken, 2009). In the present study, the density of *S. unimaculatus* was significantly greater in the sheltered reef slope than that in the rocky reef flat and no individuals were found at the seagrass bed. Furthermore, the mean FL was significant smaller in the sheltered reef slope than that in the rocky reef flat. These results suggest that, at this particular location, size-specific spatial variation in habitat use occurs for *Siganus unimaculatus*, with individuals moving from the sheltered reef slope to the rocky reef flat as they grow.

The present study also showed size-related variation in social status of the species. Most individuals less than 5.0 cm FL were found as solitary individuals and percentage of paired individuals increased for over 7 cm FL in the sheltered reef slope. In contrast, most of individuals over 14 cm FL were found as pairs. These findings are the first report for the size-specific pairing variations of the species.

The spatial variations in size might be related to the avoidance of intra- and inter-specific competition (Snover, 2008; Haywood & Kenyon, 2009). Adult pairs have distinct home ranges on the rocky reef flat and they attack other pairs. Since the smaller-sized juveniles are solitary, they would be likely to be attacked by adult pairs. Most individuals on the sheltered reef slope form pairs when they are larger than 7 cm, whereas the size of most pairs on the rocky reef flat is over 14 cm. Such size differences would be expected to make it difficult for smaller-sized pairs to successfully establish and maintain their own home ranges on the rocky reef flat. Furthermore, since juveniles of many species of coral reef fishes are found in seagrass beds (reviewed in Nagelkerken, 2009), juveniles of *S. unimaculatus* might also avoid interspecific competition for refuge space. Thus, juveniles of *S. unimaculatus* might avoid both intraspecific and interspecific competition by using the sheltered reef slope, where the density of conspecific adults and juveniles of other species are both relatively low. In addition, ontogenetic difference in habitat use often reflect changes in diet (reviewed in Nagelkerken, 2009).

The sheltered reef slope might be used for smaller-size individuals for predator avoidance. Nanami et al. (2013) have shown that the sheltered reef slope in the study site is covered by bottlebrush *Acropora* and staghorn *Acropora*, and bottlebrush *Acropora* were preferentially used by juvenile *Epinephelus ongus* (total length = less than 14 cm). Nanami et al. (2013) suggested that this was due to the fine complex structure of the bottlebrush *Acropora* as a suitable habitat for smaller-sized individuals. Such fine complex habitat structure on the sheltered reef slope might provide suitable refuge space for smaller-sized individuals of *S. unimaculatus*.

In conclusion, the present study has provided for the first time a basic understanding of the social demography of *Siganus unimaculatus* from Okinawa especially for pair formation, home range size and its overlap, agonistic behavior and spatial variation in density, size and social status.

## Supplemental Information

10.7717/peerj.1280/supp-1 Figure S1 The relationship between fork length and body mass of *Siganus unimaculatus*

10.7717/peerj.1280/supp-2 Figure S2 Inactive *Siganus unimaculatus* at nighttime The photograph was taken at Urasoko Bay, where the home range size was studied.

10.7717/peerj.1280/supp-3 Figure S3 Procedure of home range estimation of *Siganus unimaculatus* Six observations were conducted for all pairs. The enclosed bold black lines are the estimated home range for each observation. The shaded areas are estimated home range using all observations obtained just before the focal observation. The black areas are estimated home range using all six observations.

10.7717/peerj.1280/supp-4 Figure S4 Relationship between number of observations and the ‘% additional home range’ for home range size estimation ‘% additional home range’ was defined as ((*A_i_* − *A_i_* − 1)/*A_i_*-1) ×100 (Nanami & Yamada, 2008), where *A_i_* is the estimated home range by using all observations from first to *i*th observations (*i* = 2–6). The value approaches 0 with increasing number of observations.

10.7717/peerj.1280/supp-5 Figure S5 The relationship between the fork length of the smaller and larger individuals of pairs observed on the rocky reef flat and sheltered reef slope Plotting of some pairs overlapped (for detail, see ‘Fig. S5 raw data’).

10.7717/peerj.1280/supp-6 Video S1 Foraging behavior of a pair of *Siganus unimaculatus*

10.7717/peerj.1280/supp-7 Supplemental Information 1 Table 2 raw data

10.7717/peerj.1280/supp-8 Supplemental Information 2 Figure 3 Raw data

10.7717/peerj.1280/supp-9 Supplemental Information 3 Figure 5 Raw data

10.7717/peerj.1280/supp-10 Supplemental Information 4 Figure 8 raw data

10.7717/peerj.1280/supp-11 Supplemental Information 5 Figure S1 raw data

10.7717/peerj.1280/supp-12 Supplemental Information 6 Figure S4 raw data

10.7717/peerj.1280/supp-13 Supplemental Information 7 Figure S5 raw data
