# Supplementary figures and images for: Pair formation, home range, and spatial variation in density, size and social status in blotched foxface Siganus unimaculatus on an Okinawan coral reef
Source: PeerJ. 2015 Sep 24;3:e1280. doi: 10.7717/peerj.1280 (PMC4586810; doi:10.7717/peerj.1280)

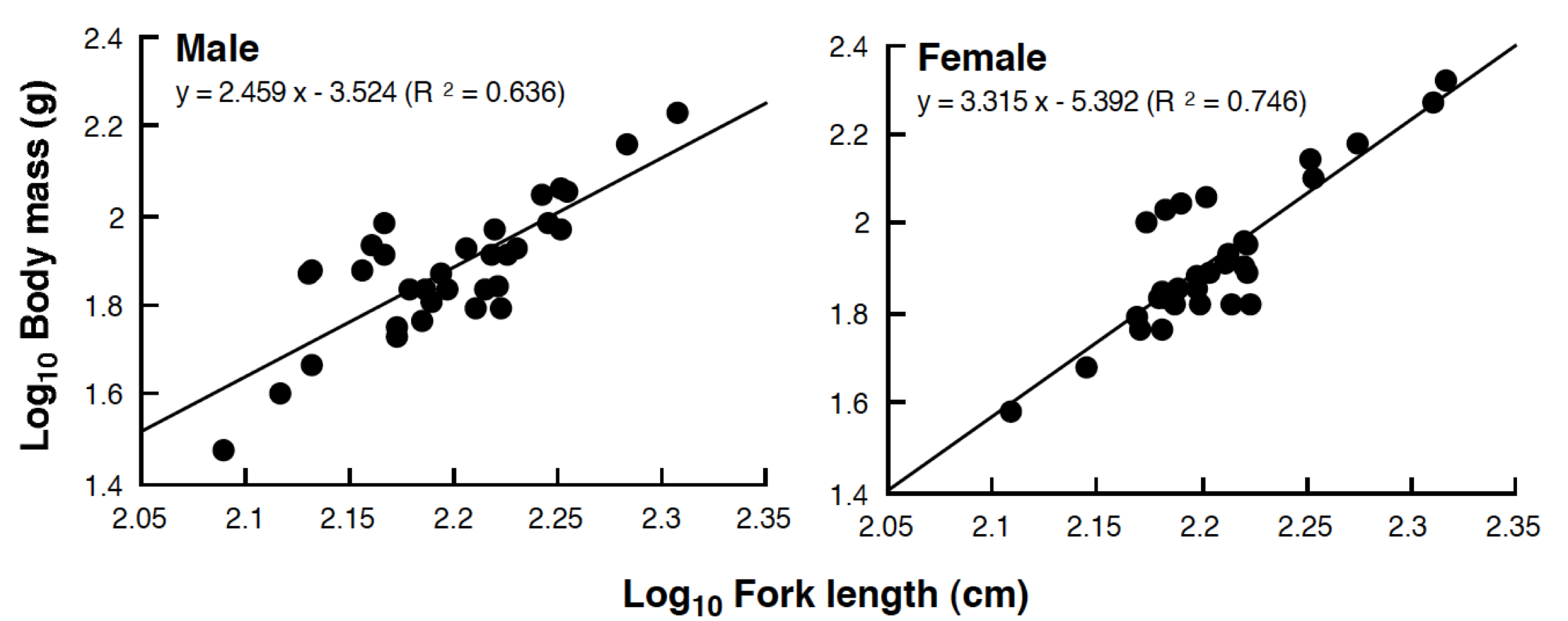

Supplement: Figure S1 [file peerj-03-1280-s001.png]

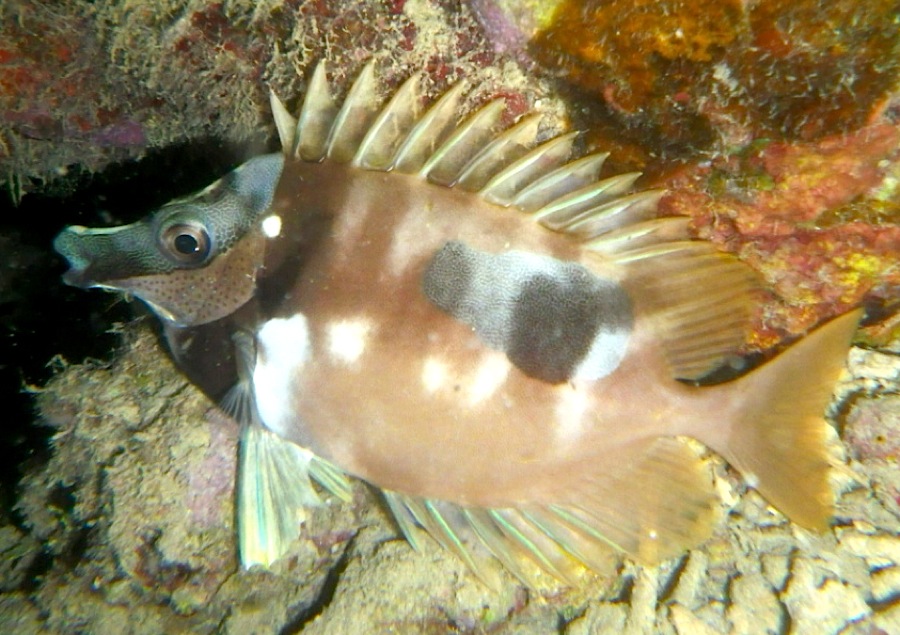

Supplement: Figure S2 — The photograph was taken at Urasoko Bay, where the home range size was studied. [file peerj-03-1280-s002.jpg]

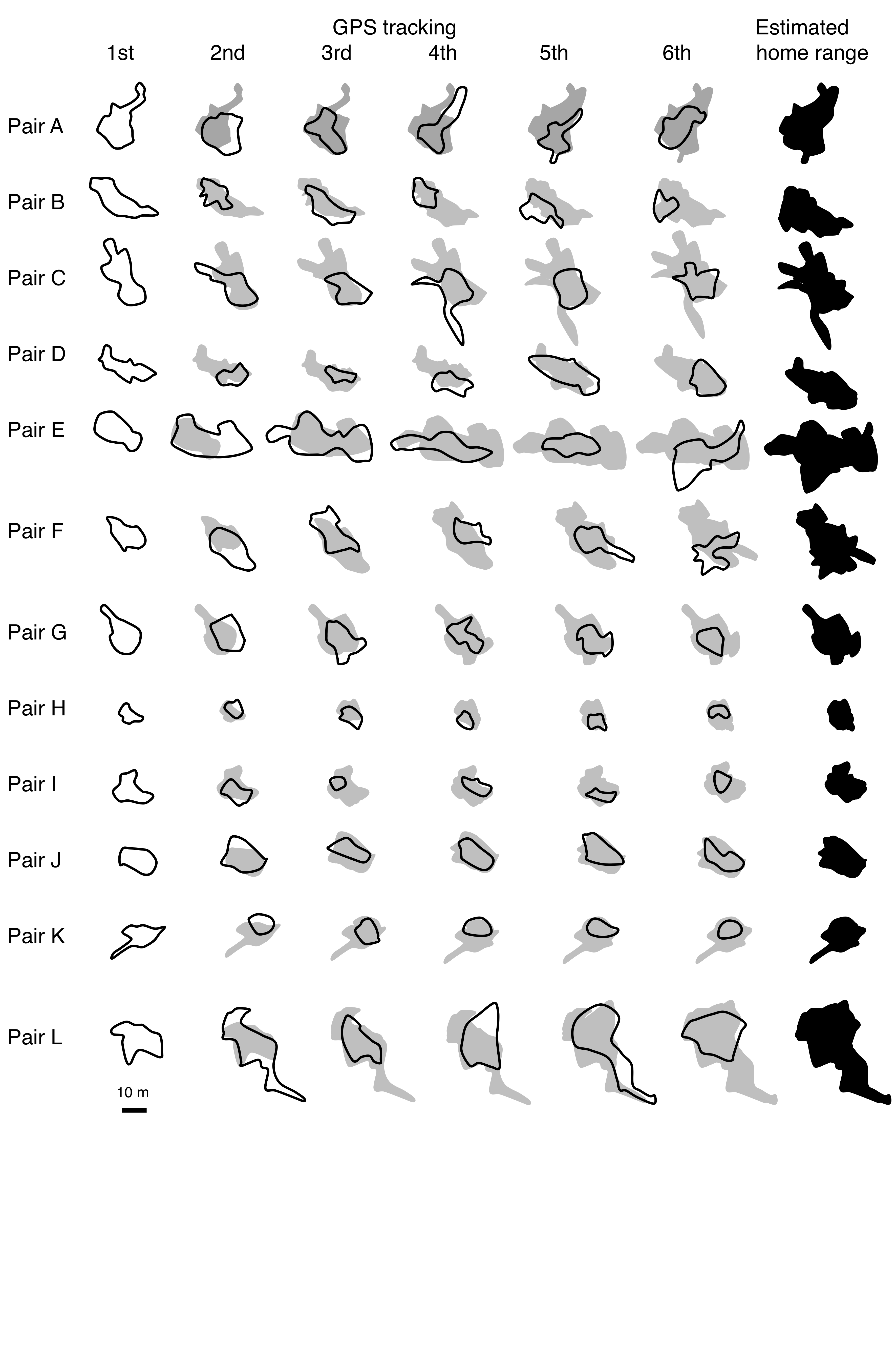

Supplement: Figure S3 — Six observations were conducted for all pairs. The enclosed bold black lines are the estimated home range for each observation. The shaded areas are estimated home range using all observations obtained just before the focal observation. The black areas are estimated home range using all six observations. [file peerj-03-1280-s003.png]

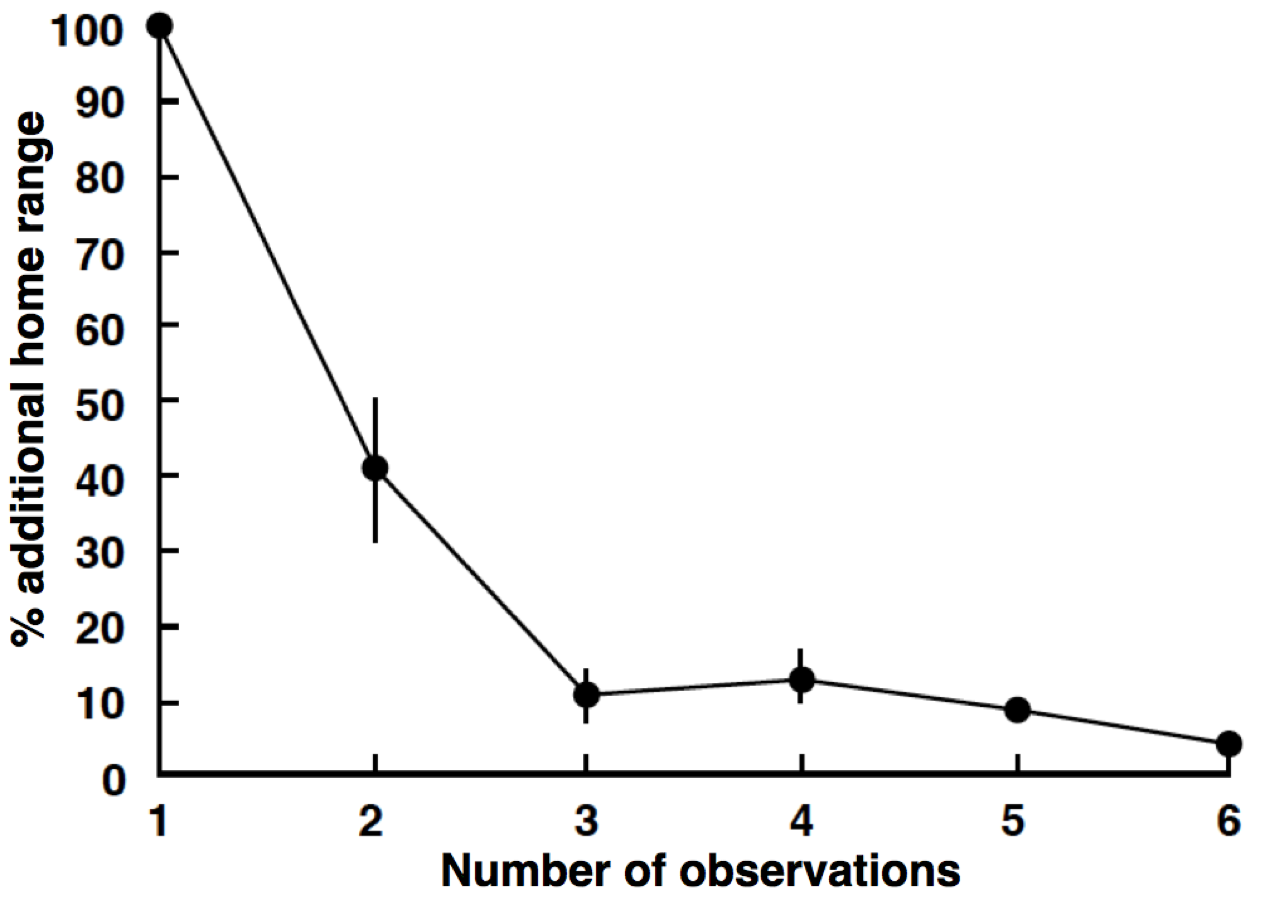

Supplement: Figure S4 — ‘% additional home range’ was defined as ((Ai − Ai − 1)/Ai-1) ×100 (Nanami & Yamada, 2008), where Ai is the estimated home range by using all observations from first to ith observations (i = 2–6). The value approaches 0 with increasing number of observations. [file peerj-03-1280-s004.png]

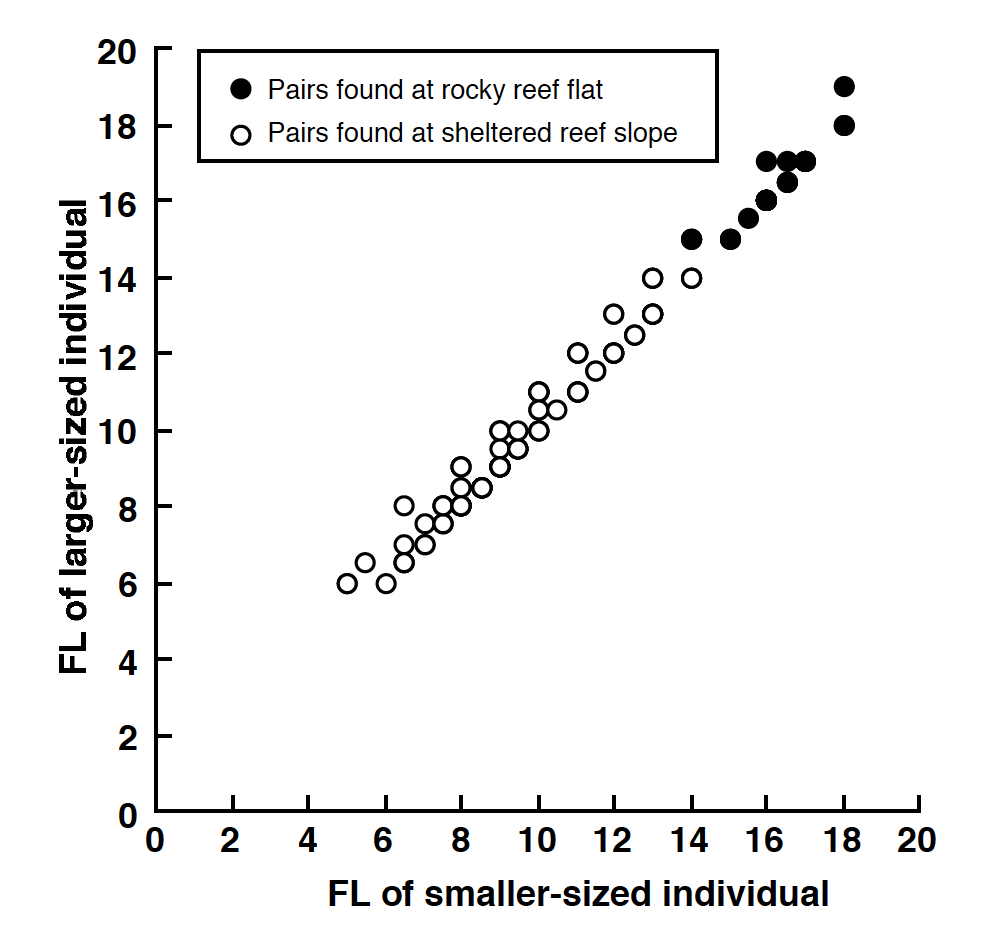

Supplement: Figure S5 — Plotting of some pairs overlapped (for detail, see ‘Fig. S5 raw data’). [file peerj-03-1280-s005.png]
